# NPCMF: Nearest Profile-based Collaborative Matrix Factorization method for predicting miRNA-disease associations

**DOI:** 10.1186/s12859-019-2956-5

**Published:** 2019-06-24

**Authors:** Ying-Lian Gao, Zhen Cui, Jin-Xing Liu, Juan Wang, Chun-Hou Zheng

**Affiliations:** 1 Library of Qufu Normal University, Qufu Normal University, Rizhao, China; 2 School of Information Science and Engineering, Qufu Normal University, Rizhao, China; 3 Co-Innovation Center for Information Supply and Assurance Technology, Anhui University, Hefei, China

**Keywords:** MiRNA-disease association prediction, Nearest profile, Gaussian interaction profile, Matrix factorization

## Abstract

**Background:**

Predicting meaningful miRNA-disease associations (MDAs) is costly. Therefore, an increasing number of researchers are beginning to focus on methods to predict potential MDAs. Thus, prediction methods with improved accuracy are under development. An efficient computational method is proposed to be crucial for predicting novel MDAs. For improved experimental productivity, large biological datasets are used by researchers. Although there are many effective and feasible methods to predict potential MDAs, the possibility remains that these methods are flawed.

**Results:**

A simple and effective method, known as Nearest Profile-based Collaborative Matrix Factorization (NPCMF), is proposed to identify novel MDAs. The nearest profile is introduced to our method to achieve the highest AUC value compared with other advanced methods. For some miRNAs and diseases without any association, we use the nearest neighbour information to complete the prediction.

**Conclusions:**

To evaluate the performance of our method, five-fold cross-validation is used to calculate the AUC value. At the same time, three disease cases, gastric neoplasms, rectal neoplasms and colonic neoplasms, are used to predict novel MDAs on a gold-standard dataset. We predict the vast majority of known MDAs and some novel MDAs. Finally, the prediction accuracy of our method is determined to be better than that of other existing methods. Thus, the proposed prediction model can obtain reliable experimental results.

## Background

MicroRNAs (miRNAs) are small non-coding RNAs whose length is generally 19 to 25 nt [1, 2]. In general, miRNAs regulate the expression of mRNA targets through a series of biological processes. However, the imbalance of miRNAs may have a serious impact on humans. Therefore, identifying novel miRNA-disease associations is important for treating complex genetic diseases [3, 4]. The first miRNA, lin-4, was discovered in 1993. It is worth noting that lin-4 is not the same as a conventional protein-coding gene; instead, lin-4 encodes a 22-nt regulatory RNA [5, 6]. In 2000, the second miRNA, let-7, was discovered by researchers [7]. Since then, thousands of miRNAs have been discovered by biologists through a variety of biological and medical approaches. More than 2000 human miRNAs have been detected. Moreover, the latest version of the miRNA database miRBase contains 38,589 entries.

Recently, many biologists and medical scientists have found that miRNAs play an important role in different biological processes. In addition, an increasing number of miRNAs have been shown to be associated with cancer and other human diseases. For example, invasion and migration of breast cancer cells are inhibited by mir-340 by targeting the oncoprotein c-Met [8]. In addition, by targeting Cdc42 and Cdk6, mir137 inhibits the proliferation of lung cancer cells [9]. The progression of head and neck carcinomas is promoted by miR-211 through the target TGFβR2 [10]. Moreover, in every paediatric brain tumour type, mir-25, mir-129, and mir-142 are differentially expressed [11]. By identifying unknown potential miRNA-disease associations, the molecular mechanisms and pathogenesis of the disease can be elucidated.

In recent years, many researchers have employed computational methods associated with biomolecules and diseases [12–15]. In previous studies, an important assumption is that miRNAs with similar functions are more likely to be associated with diseases with similar phenotypes [16]. In other words, miRNAs with similar functions may be associated with the same disease. Increasingly effective methods and models are proposed for identifying novel miRNA-disease associations (MDAs). Chen et al. proposed a computational model named RLSMDA (Regularized Least Squares miRNA-Disease Association) based on semi-supervised learning [17]. In this way, the problem of using negative MDAs is overcome. However, this semi-supervised model is not perfect for the optimization of some parameters. Importantly, classifiers from the miRNA space and disease space are difficult to combine to predict novel MDAs. Chen et al. proposed a Path-Based MiRNA-Disease Association (PBMDA) prediction model [15]. Specifically, a depth-first search algorithm is used to predict novel MDAs on a heterogeneous graph consisting of three interlinked sub-graphs. Chen et al. proposed a computational model named BNPMDA (Bipartite Network Projection for MiRNA-Disease Association) to obtain some valuable and reliable results [18]. The degree of preference between miRNA and disease is first described, then agglomerative hierarchical clustering is used, and finally, the BNPMDA method is implemented to predict potential MDAs. Jiang et al. constructed a model based on hypergeometric distribution through miRNA functional similarity, disease similarity and known MDA networks [19]. Then, these researchers analysed the actual effect in the prediction model. However, the shortcoming of this model is the excessive dependence on neighbouring miRNA data [20]. Chen et al. proposed a computational method to predict novel MDAs by using Laplacian regularized sparse subspace learning, and the accuracy of the prediction is improved [21]. Laplacian regularization is used to preserve the local structures. The strength of dimensionality reduction makes it easy to experiment with higher-dimensional datasets. Shi et al. proposed a computational method to predict novel MDAs by performing a random walk algorithm [22]. Protein-protein interactions (PPIs), miRNA-target interactions and disease-gene associations were used to discover potential MDAs. This model is reliable, but there are still some shortcomings. The model strongly depended on the miRNA-target interactions. Therefore, the final experimental results may have a high false positive rate or a high false negative rate [23]. Considering this disadvantage, Chen et al. developed a new method to solve this problem. The Random Walk with Restart for MiRNA-Disease Association (RWRMDA) model was used to map all miRNAs to a miRNA functional similarity network [24]. Mork et al. considered the protein information and proposed the miRPD method [25]. The method relies on protein-disease associations and protein-miRNA associations to predict novel miRNAs and disease-related proteins. Chen et al. proposed an effective method, Heterogeneous Graph Inference MiRNA-Disease Association (HGIMDA), to predict novel MDAs [26]. In this method, Gaussian interaction profile (GIP) kernel similarity for diseases and miRNAs are integrated into the computational model. According to the final experimental results, this method improves the prediction accuracy. Chen et al. also proposed an effective method, Matrix Decomposition and Heterogeneous Graph Inference (MDHGI), to predict novel MDAs [14]. Among these approaches, the largest contribution is the combination of matrix decomposition and heterogeneous graph inference to predict new MDAs. In addition, Chen et al. proposed a method called inductive matrix completion [13]. The main measure is to complete the missing miRNA-disease association. Xuan et al. proposed an HDMP method based on weighting k-nearest neighbours [27]. Moreover, the semantic similarity and phenotypic similarity of the diseases were used to participate in the calculation of the functional similarity matrix of miRNAs. In contrast to previous studies, miRNAs of the same cluster have higher weights; therefore, they have the greatest potential to be associated with similar diseases when calculating the miRNA functional similarity matrix. Based on Xuan et al.’s method, Chen et al. proposed an improved method called RKNNMDA to identify potential MDAs [28]. Later, a valuable model named Matrix Completion for MiRNA-Disease Association prediction (MCMDA) was proposed by Li et al. [29]. However, this approach has certain limitations for new diseases and new miRNAs. These limitations lead to inaccuracies in the prediction results. Chen et al. developed a computational model named Ensemble Learning and Link Prediction for MiRNA-Disease Association (ELLPMDA) to identify potential MDAs [30]. Integrated similarity networks and integrated learning were used to predict novel MDAs. At the same time, this method is one of the more advanced methods. Chen et al. compiled the most advanced 20 prediction models to illustrate the importance of MDA prediction. Computational models have become an important means for novel MDA identification. The most important point is that the review can be inspired by more researchers [31].

In this paper, a simple but effective Nearest Profile-based Collaborative Matrix Factorization (NPCMF) method is proposed. This computational method can identify potential MDAs based on known MDAs. More importantly, unlike traditional matrix factorization models, considering that a new miRNA or a new disease is affected by their neighbour information when predicted, the nearest profile (NP) [32] is introduced to the CMF. The benefit of NP is that the nearest neighbour information for miRNA and disease is taken into account. The NP performs prediction through relatively reliable similarity functions. More precisely, the association profile of a new miRNA or disease is predicted using its similarities to other miRNAs or diseases, respectively; a new miRNA is one that has no known diseases, and similarly, a new disease is one that has no known interactions with any miRNAs. Notably, the existence of a large number of missing associations will have a negative impact on the final predictions. Weighted K Nearest Known Neighbours (WKNKN) is used as a pre-processing step to solve this problem [33]. Meanwhile, five-fold cross-validation is performed to evaluate our experimental results. In addition, a simulation experiment is conducted to predict novel MDAs. Finally, the results demonstrate that our proposed method NPCMF is superior to other advanced methods.

The rest of this paper is organized as follows. Section 2 is first described, including our final experimental results and the gold-standard dataset used in this study. Section 3 contains the corresponding discussion. Section 4 contains conclusions for the full paper. Finally, Section 5 outlines our proposed method, specific solution steps and iterative processes.

## Results

### MDA dataset

The datasets used in the experiments were obtained from the human miRNA-disease database (HMDD), including 383 diseases, 495 miRNAs and 5430 human miRNA-disease associations [20]. The HMDD, which is a well-known bioinformatics database, has collected thousands of miRNA-disease association pairs. Table 1 lists the specific information for the dataset.

**Table 1:** MiRNAs, diseases, and associations in Gold Standard Dataset

Datasets	MiRNAs	Diseases	Associations
Gold Standard Dataset	495	383	5430

In addition, the dataset contains three matrices: **Y** ∈ ℝ^*n* × *m*^, **S**_m_ ∈ ℝ^*n* × *n*^ and **S**_d_ ∈ ℝ^*m* × *m*^. The matrix **Y** is an adjacency matrix that is used to describe the associations between miRNAs and diseases. There are *n* miRNAs as rows and *m* diseases as columns. If miRNA *M*(*i*) is associated with disease *d*(*j*), the entity **Y**(*M*(*i*), *d*(*j*)) is 1; otherwise, it is 0. Moreover, this dataset is still a gold-standard dataset. The matrix **Y** is expressed as follows:

1 $$ \mathbf{Y}\left(M(i),d(j)\right)=\left\{\begin{array}{l}1,\kern1.6em \mathrm{if}\ \mathrm{miRNA}\ M(i)\ \mathrm{associated}\ \mathrm{with}\ \mathrm{disease}\ d(j),\\ {}0,\kern1.4em \mathrm{otherwise}.\end{array}\right. $$

### Performance evaluation metrics

To evaluate our approach, five-fold cross-validation is conducted 100 times for each method. The known MDA dataset is randomly divided into 5 subsets, 4 of which are used as training sets, and the remaining subset is used as a testing set. It is worth noting that in our approach, WKNKN is used to eliminate unknown missing values. At the same time, the advantage is that the accuracy of the prediction can be improved to some extent.

In previous studies, the area under the curve (AUC) value is a reliable indicator of the evaluation method. Therefore, the AUC value is also used in this study. The area under the receiver operating characteristic (ROC) curve is considered to be the AUC. In general, the value of this area will not be greater than 1. The AUC values between 0.5 and 1 are reasonable. If the AUC is less than 0.5, the predicted results will be meaningless. In general, the ROC curve can be described in terms of true positive rate (TFR, sensitivity) and false positive rate (FPR, 1-specificity). Thus, sensitivity and specificity (SPEC) can be expressed as follows:

2 $$ Sensitivity=\frac{TP}{TP+ FN}, $$

3 $$ Specificity=\frac{TN}{N}=\frac{TN}{TN+ FP}, $$

where, according to the classification of the classifier, *TP* is the number of positive samples, *FN* is the number of false negative samples, and *N* is the number of negative samples. Similarly, *TN* is the number of negative samples, and *FP* is the number of false positive samples.

The MDA pairs are randomly removed in the input matrix **Y** before performing cross-validation. This method is called CV-p (Cross-Validation pairs). Moreover, the purpose is to overcome the difficulty of prediction and accurately evaluate our method.

### Comparison with other methods

In this study, the NPCMF method was compared with other advanced methods, CMF [34], HDMP [35], WBSMDA [36], HAMDA [37], and ELLPMDA [30]. Table 2 lists the experimental results with CV-p. In Table 2, the final experimental results are expressed as the average of 100 five-fold cross-validation. It is worth noting that AUC is known to be insensitive to skewed class distributions [38]. Considering that the dataset used in this paper is highly unbalanced, there are more negative factors than positive ones. Thus, AUC is a fair and reasonable evaluation indicator for all methods.

**Table 2:** AUC results of cross validation experiments

Methods	Gold Standard Dataset
WBSMDA	0.8185 (0.0009)
HDMP	0.8342 (0.0010)
CMF	0.8697 (0.0011)
HAMDA	0.8965 (0.0012)
ELLPMDA	0.9193 (0.0002)
NPCMF	0.9429 (0.0011)

**Fig. 1:**
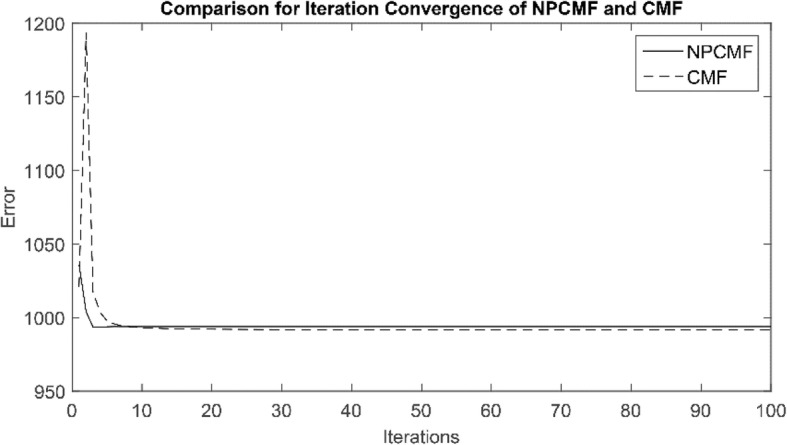
Comparison of convergence about NPCMF and CMF. Compared with the CMF, the NPCMF converges the fastest

**Fig. 2:**
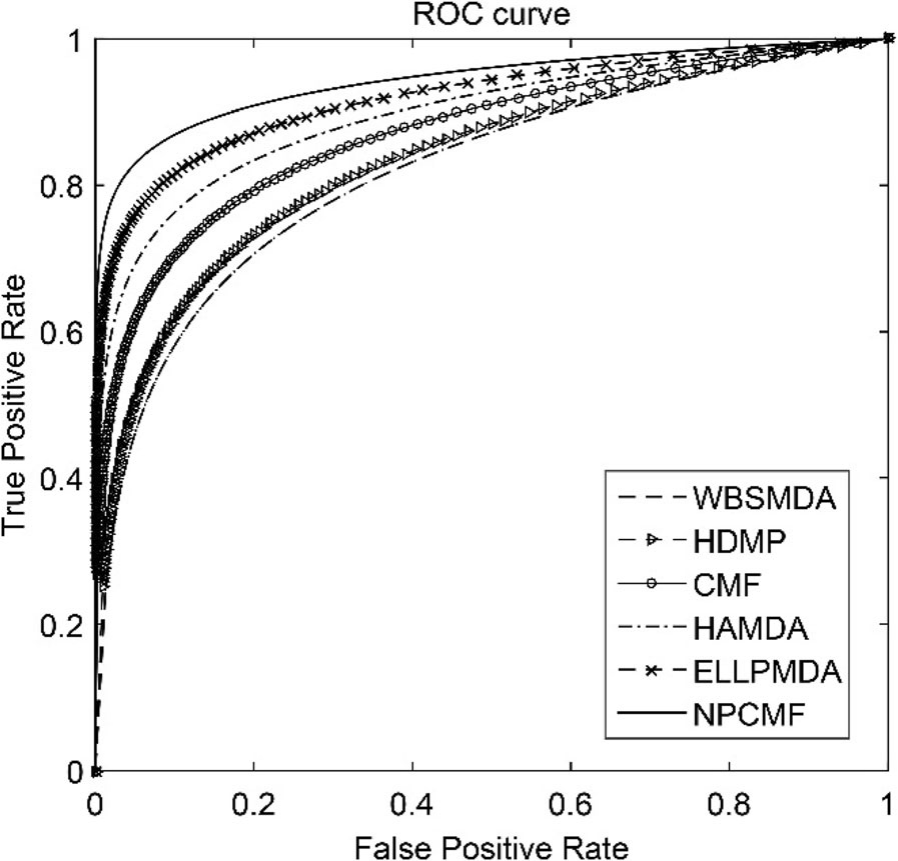
The ROC curve for each method in a 5-fold cross validation experiment

As listed in Table 2, the average AUCs of WBSMDA, HDMP, CMF, HAMDA, ELLPMDA, and NPCMF on the gold-standard dataset are 0.8185 ± 0.0009, 0.8342 ± 0.001, 0.8697 ± 0.0011, 0.8965 ± 0.0012, 0.9193 ± 0.0002 and 0.9429 ± 0.0011, respectively. The best value is in bold. Standard deviations are given in parentheses. From the above statistical results, our method achieved the highest AUC value, which was 12.46, 10.89, 7.34, 4.66, and 2.36% higher than WBSMDA, HDMP, CMF, HAMDA, and ELLPMDA, respectively. Compared with the CMF method, our method NPCMF has the best convergence. Furthermore, as shown in Fig. 1, the convergence analysis of CMF and NPCMF is shown by performing 100 iterations. Therefore, based on the above results, our proposed method is better than other existing advanced methods. Thus, the NPCMF method has proven to be effective and reliable. As shown in Fig. 2, in the five-fold cross-validation experiment, the performance of each method can be demonstrated using the ROC curve.

### Sensitivity analysis from WKNKN

**Fig. 3:**
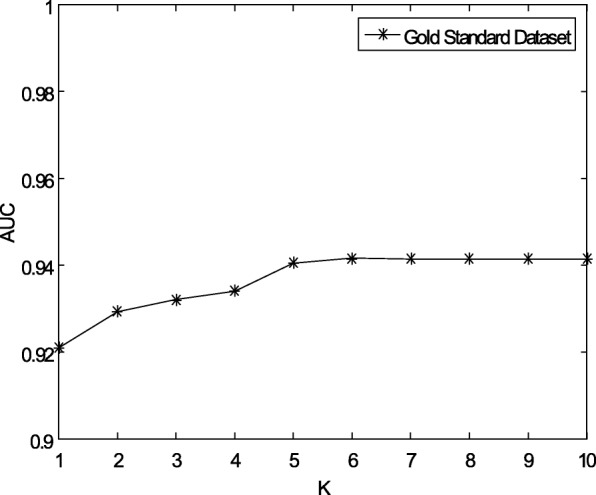
Sensitivity analysis for *K* under CV-p

**Fig. 4:**
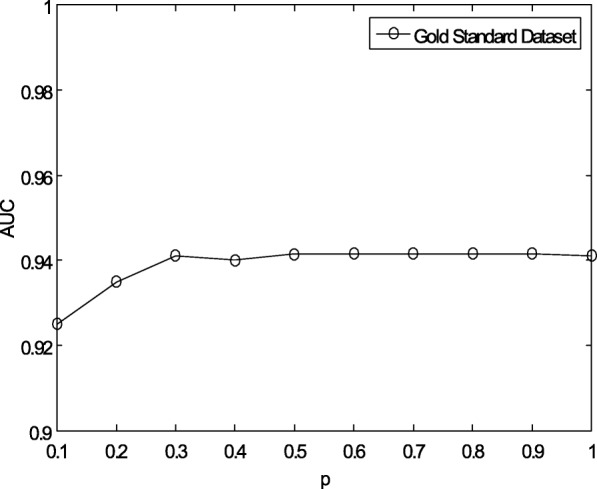
Sensitivity analysis for *p* under CV-p

Considering that there are some missing unknown associations in the matrix **Y**, WKNKN pre-processing is used to minimize the error. *K* represents the number of nearest known neighbours. *p* represents a decay term where *p* ≤ 1. These two parameters will be fixed to the optimal value before performing our method NPCMF. The sensitivities regarding *K* and *p* are represented by Figs. 3 and 4, respectively. The AUC tends to be stable when *K* = 5 and *p* = 0.7.

### Comprehensive prediction for novel MDAs

A simulation experiment is conducted in this subsection. The simulation is conducted to obtain the final prediction score matrix. The specific process is divided into four steps. The first step is to execute our method; then, the two matrices **A** and **B** are obtained. The second step is to multiply **A** and **B** to obtain a predicted score matrix. The third step is to compare the predicted score matrix with the original MDAs matrix **Y** and the associations whose predicted score changes are filtered and sorted. The fourth step is to use the existing database to verify that our predicted associations are confirmed. Our method is applied to three disease cases, gastric neoplasms, rectal neoplasms and colonic neoplasms. These three diseases are more common among humans. Many miRNAs are closely related to these three diseases. Therefore, the final prediction results are more universal. In addition, the novel MDAs are validated by two popular miRNA disease databases, dbDEMC and miR2Disease.

The first case is gastric neoplasms. Despite a declining incidence [39], gastric neoplasms are a major cause of cancer death worldwide. Gonzalez et al. observed that gastric neoplasms constitute the second most frequent cancer in the world and the fourth most frequent cancer in Europe [40]. More information about the disease is published in http://www.omim.org/entry/613659. In the dataset used in the experiment, there are five MDAs associated with gastric neoplasms. After the simulation experiment is performed, three known associations are successfully predicted. At the same time, seven novel MDAs are predicted. More importantly, five of the seven novel MDAs have been confirmed by dbDEMC or miR2Disease. It is worth noting that miR-214 is confirmed by both databases. For example, in 2011, when Oh et al. identified the biological validity of oncogenic miRNA microarray data for gastric neoplasms, miR-214 in GC-2 miRNAs was observed to be significantly upregulated [41]. In 2013, Lim et al. also found that miR-214 is overexpressed in patients with gastric neoplasms compared with normal subjects [42]. It is worth noting that although both miR-30b and miR-296 are not confirmed by these two databases, they are still strongly associated with gastric neoplasms. Table 3 lists the detailed experimental results. The known associations are in bold.

**Table 3:** Predicted MiRNAs for Gastric Neoplasms

Rank	miRNA	Evidence
**1**	**hsa-mir-1**	known
**2**	**hsa-mir-23a**	known
**3**	**hsa-mir-148a**	known
4	hsa-mir-214	dbDEMC; miR2Disease
5	hsa-mir-30b	Unconfirmed
6	hsa-mir-145	dbDEMC
7	hsa-mir-296	Unconfirmed
8	hsa-mir-199a	miR2Disease
9	hsa-mir-23b	dbDEMC
10	hsa-mir-96	dbDEMC

The second case is rectal neoplasms. Fourteen known miRNAs were successfully predicted. Because there are more miRNAs associated with rectal neoplasms, we only selected the top 20 miRNAs with the highest correlation with rectal neoplasms. In Table 4, the miRNAs are arranged in descending order of the association score. Among the new miRNAs that are predicted, the fifteenth miRNA, miR-196a, has the highest association score. Regarding miR-196a, it was confirmed in the previous literature that it is associated with lymphoma [43]. Other researchers have found that miR-196a is associated with prostate neoplasms [44]. Although the predicted novel MDAs are not confirmed by dbDEMC or miR2Disease, according to our experimental results, these MDAs are closely related to rectal neoplasms. Table 4 lists the detailed experimental results. The known associations are in bold.

**Table 4:** Predicted MiRNAs for Rectal Neoplasms

Rank	miRNA	Evidence
**1**	**hsa-mir-21**	known
**2**	**hsa-mir-145**	known
**3**	**hsa-mir-125b**	known
**4**	**hsa-mir-16**	known
**5**	**hsa-mir-7**	known
**6**	**hsa-mir-153**	known
**7**	**hsa-mir-1224**	known
**8**	**hsa-mir-137**	known
**9**	**hsa-mir-622**	known
**10**	**hsa-mir-630**	known
**11**	**hsa-mir-720**	known
**12**	**hsa-mir-590**	known
**13**	**hsa-mir-765**	known
**14**	**hsa-mir-1471**	known
15	hsa**-**mir-196a	Unconfirmed
16	hsa**-**mir-203	Unconfirmed
17	hsa**-**mir-196b	Unconfirmed
18	hsa**-**mir-132	Unconfirmed
19	hsa**-**mir-375	Unconfirmed
20	hsa**-**mir-199b	Unconfirmed

The third case is colonic neoplasms. From the gold-standard dataset used in the experiment, there are more than 50 miRNAs related to colonic neoplasms; therefore, the top 50 are selected as the final prediction results according to the association score. Thirty known miRNAs are successfully predicted, and 20 new miRNAs are predicted. Of the 20 predicted new miRNAs, 12 are confirmed by dbDEMC and 8 are unconfirmed. For example, in 2009, Sarver et al. found that miR-520 g was overexpressed in patients with colonic neoplasms compared with normal people according to a reliable biological experiment [43]. These researchers also found that miR-204, miR-206 and miR-215 tend to be negatively expressed in colonic neoplasm patients. In addition, some unconfirmed miRNAs are sorted in descending order of association scores, including miR-144, miR-515, miR-211, miR-525, miR-219, miR-339, miR-124 and miR-340. Table 5 lists the detailed experimental results. The known associations are in bold.

**Table 5:** Predicted MiRNAs for Colonic Neoplasms

Rank	miRNA	Evidence	Rank	miRNA	Evidence
**1**	**hsa-mir-146a**	known	**26**	**hsa-let-7d**	known
**2**	**hsa-mir-18a**	known	**27**	**hsa-mir-30a**	known
**3**	**hsa-mir-29a**	known	**28**	**hsa-mir-22**	known
**4**	**hsa-mir-106b**	known	**29**	**hsa-mir-200c**	known
**5**	**hsa-mir-92a**	known	**30**	**hsa-mir-191**	known
**6**	**hsa-mir-32**	known	31	hsa-mir-520 g	dbDEMC
**7**	**hsa-mir-200b**	known	32	hsa-mir-204	dbDEMC
**8**	**hsa-mir-29b**	known	33	hsa-mir-206	dbDEMC
**9**	**hsa-mir-10b**	known	34	hsa-mir-215	dbDEMC
**10**	**hsa-mir-15a**	known	35	hsa-mir-491	dbDEMC
**11**	**hsa-let-7c**	known	36	hsa-mir-144	Unconfirmed
**12**	**hsa-mir-142**	known	37	hsa-mir-515	Unconfirmed
**13**	**hsa-mir-132**	known	38	hsa-mir-153	dbDEMC
**14**	**hsa-mir-155**	known	39	hsa-mir-211	Unconfirmed
**15**	**hsa-mir-101**	known	40	hsa-mir-525	Unconfirmed
**16**	**hsa-mir-19a**	known	41	hsa-mir-219	Unconfirmed
**17**	**hsa-let-7i**	known	42	hsa-mir-526b	dbDEMC
**18**	**hsa-mir-133b**	known	43	hsa-mir-507	dbDEMC
**19**	**hsa-mir-16**	known	44	hsa-mir-523	dbDEMC
**20**	**hsa-mir-34a**	known	45	hsa-mir-520f	dbDEMC
**21**	**hsa-mir-31**	known	46	hsa-mir-520e	dbDEMC
**22**	**hsa-mir-125a**	known	47	hsa-mir-339	Unconfirmed
**23**	**hsa-mir-141**	known	48	hsa-mir-124	Unconfirmed
**24**	**hsa-mir-17**	known	49	hsa-mir-381	dbDEMC
**25**	**hsa-mir-1**	known	50	hsa-mir-340	Unconfirmed

## Discussion

Based on the above experimental results, our proposed model NPCMF is superior to the most advanced methods overall. Moreover, although CMF is not as good as NPCMF, it has also achieved good experimental results. It is worth noting that our greatest contribution is to calculate the NP information for each disease and each miRNA to help predict potential MDAs. The shortcomings of CMF are that for new miRNAs and new diseases, the CMF method is unpredictable. However, NPCMF can achieve the prediction of new miRNAs and new diseases by using each miRNA and the nearest neighbour of the disease. Therefore, it is precisely because of the introduction of NP information that some novel MDAs can be predicted. By using NP information, we can obtain the best AUC value. Of course, this finding does not prove that NPCMF has no defects. One of the most obvious drawbacks for NPCMF is that excessive NP information is introduced, which may add additional noise while reducing prediction accuracy.

## Conclusions

In this paper, a novel method based on nearest profile collaborative matrix factorization is developed for predicting novel MDAs. When novel MDAs are predicted, the nearest neighbour information for miRNAs and diseases is fully considered. In addition, incorporating the Gaussian interaction profile kernels of miRNAs and diseases also contributed to the improvement of prediction performance. The AUC value is used as a reliable indicator to evaluate our method. In addition, due to technical limitations, we have not used the latest version of the dataset, such as HMDD V3.0; therefore, we will attempt to use the latest dataset for future experiments.

In the future, more effective methods may be used to predict new MDAs. More differentially expressed miRNAs associated with the disease will be identified. At the same time, increasing numbers of valuable datasets are being published by online bioinformatics databases. Thus, more datasets can be tested by researchers. Importantly, NPCMF may be helpful for novel MDA prediction and relevant miRNA research from computational biology.

## Methods

Our goal is to develop a matrix factorization method that can predict novel MDAs based on known MDAs. First, a matrix factorization model is constructed to represent the correlation between miRNAs and diseases. Next, the Gaussian interaction profile kernels of miRNA and disease are expressed as their network information. Then, the nearest profile of miRNAs and diseases are obtained. Finally, a prediction score matrix is obtained by multiplying two low rank matrices.

### MiRNA functional similarity

Wang et al. developed a method named MISIM for calculating the similarity scores of miRNA functions [45]. Moreover, the dataset that we used is downloaded from the website http://www.cuilab.cn/files/images/cuilab/misim.zip. Then, matrix **S**_m_ represents the functional similarity matrix of the miRNAs. Since the self-similarity of a miRNA is 1, in the matrix **S**_m_, the elements on the diagonal are all 1.

### Disease semantic similarity

In previous studies, directed acyclic graphs (DAGs) have been used by many researchers to describe diseases. From the National Library of Medicine (http://www.nlm.nih.Gov/), a variety of disease relationships based on the disease DAG can be obtained from the MeSH descriptor of Category C. DAG(*DD*) = (*d*, *T*(*DD*), *E*(*DD*)) is used to describe disease *DD*. *T*(*DD*) is the node set and *E*(*DD*) is the corresponding link set. The *DD* in DAG(*DD*) formula is defined as

4 $$ DV1(DD)=\sum \limits_{d\in T(DD)}D{1}_{DD}(d), $$

5 $$ D{1}_{\mathrm{DD}}(d)=\left\{\begin{array}{l}1\kern15.30002em if\kern0.3em d= DD,\\ {}\max \left\{\varDelta \ast D{1}_{DD}\left({d}^{\hbox{'}}\right)\left|{d}^{\hbox{'}}\in children\right. of\kern0.1em d\right\}\kern0.5em if\kern0.3em d\ne DD,\end{array}\right. $$

where *Δ* represents the semantic contribution factor. In this work, based on previous literature [45], the value of *Δ* is set to 0.5.

In addition, matrix **S**_d_ represents the semantic similarity matrix of the disease. Similarly, in the matrix **S**_d_, the elements on the diagonal are all 1. It is worth noting that if the two diseases *d*(*i*) and *d*(*j*) have a larger common part of the DAGs, these two diseases will have higher semantic similarity values. The semantic similarity score between two diseases is defined as follows:

6 $$ {\mathbf{S}}_{\mathrm{d}}\left(d(i),d(j)\right)=\frac{\sum_{t\in T\left(d(i)\right)\cap T\left(d(j)\right)}\left(D{1}_{d(i)}(t)+D{1}_{d(j)}(t)\right)}{DV1\left(d(i)\right)+ DV1\left(d(j)\right)}. $$

### Gaussian interaction profile kernel similarity

**Fig. 5:**
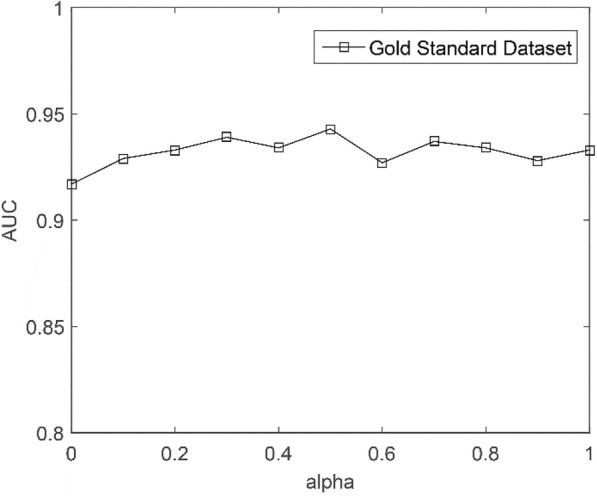
Sensitivity analysis for *α* under CV-p

The method is based on the following assumption. The topological structure of the known MDA network is represented by Gaussian interaction profile kernel similarity [46]. *M*(*i*) and *M*(*j*) are two miRNAs, and *d*(*i*) and *d*(*j*) are two diseases. Therefore, the network similarity calculations can be written as

7 $$ {GIP}_{miRNA}\left({M}_{i,}{M}_j\right)=\exp \left(-\gamma {\left\Vert \mathbf{Y}\left({M}_i\right)-\mathbf{Y}\left({M}_j\right)\right\Vert}^2\right), $$

8 $$ {GIP}_{disease}\left({d}_{i,}{d}_j\right)=\exp \left(-\gamma {\left\Vert \mathbf{Y}\left({d}_i\right)-\mathbf{Y}\left({d}_j\right)\right\Vert}^2\right), $$

where *γ* is expressed as a parameter that adjusts the bandwidth of the kernel. In principle, the setting of *γ* should be implemented by cross-validation, but according to a previous study [47], *γ* is simply set to 1. In addition, the interaction profiles of *M*_*i*_ and *M*_*j*_ can be represented as **Y**(*M*_*i*_) and **Y**(*M*_*j*_), respectively. Similarly, the interaction profiles of *d*_*i*_ and *d*_*j*_ can be represented as **Y**(*d*_*i*_) and **Y**(*d*_*j*_), respectively. Thus, the miRNA network similarity matrix can be combined by **S**_m_ into **K**_m_, and the disease network similarity matrix can be combined by **S**_d_ into **K**_d_. The calculation formulas are as follows:

9 $$ {\mathbf{K}}_{\mathrm{m}}=\alpha {\mathbf{S}}_{\mathrm{m}}+\left(1-\alpha \right){\mathbf{GIP}}_{\mathrm{m}}, $$

10 $$ {\mathbf{K}}_{\mathrm{d}}=\alpha {\mathbf{S}}_{\mathrm{d}}+\left(1-\alpha \right){\mathbf{GIP}}_{\mathrm{d}}, $$

where *α* ∈ [0, 1] is an adjustable parameter. We perform a sensitivity analysis on *α*. When *α* = 0.5, the highest AUC value can be obtained. Figure 5 shows the sensitivity analysis for *α*. **K**_m_ is a miRNA kernel matrix, which represents a linear combination of the miRNA functional similarity matrix **S**_m_ and the miRNA network similarity matrix **GIP**_m_. Similarly, **K**_d_ is similar to **K**_m_. **K**_d_ is a disease kernel matrix. In each cross-validation, we recalculate the miRNA Gaussian similarity and disease Gaussian similarity. Specifically, the miRNA Gaussian similarity matrix and the disease Gaussian similarity matrix are obtained from a known MDA matrix. Therefore, we ensure that the Gaussian similarity is recalculated each time the cross-validation is performed so that the Gaussian similarity correctly reflects the characteristics of the MDA matrix.

### NPCMF for MDA prediction

The traditional CMF is a reliable method for predicting novel MDAs [34]. Collaborative filtering is introduced to CMF. The objective function of CMF is defined as

11 $$ {\min}_{\mathbf{A},\mathbf{B}}={\left\Vert \mathbf{Y}-{\mathbf{A}\mathbf{B}}^{\mathrm{T}}\right\Vert}_F^2+{\lambda}_l\left({\left\Vert \mathbf{A}\right\Vert}_F^2+{\left\Vert \mathbf{B}\right\Vert}_F^2\right)+{\lambda}_d{\left\Vert {\mathbf{S}}_{\mathrm{m}}-{\mathbf{A}\mathbf{A}}^{\mathrm{T}}\right\Vert}_F^2+{\lambda}_t{\left\Vert {\mathbf{S}}_{\mathrm{d}}-{\mathbf{BB}}^{\mathrm{T}}\right\Vert}_F^2, $$

where ‖⋅‖_*F*_ is the Frobenius norm, and *λ*_*l*_, *λ*_*d*_ and *λ*_*t*_ are non-negative parameters. It is worth noting that the three parameters are set on the training set by performing cross-validation. A grid search is used to obtain the optimal parameters from these values: *λ*_*l*_ ∈ {2^−2^, 2^−1^, 2^0^, 2^1^}, *λ*_*d*_/*λ*_*l*_ ∈ {0, 10^−4^, 10^−3^, 10^−2^, 10^−1^}. The MDA matrix **Y** is decomposed into two matrices **A** and **B**, where **AB**^T^ ≈ **Y**. The NPCMF method uses regularization terms to request that the potential feature vectors of similar miRNAs and similar diseases are similar, and the potential feature vectors of dissimilar miRNAs and dissimilar diseases are dissimilar, respectively [33]. In this instance, **S**_m_ ≈ **AA**^T^ and **S**_d_ ≈ **BB**^T^.

However, the CMF method ignores the network information of miRNAs and diseases. Therefore, GIP is introduced to the CMF [48]. Therefore, **K**_m_ and **K**_d_ are substituted into the objective function and written as

12 $$ {\displaystyle \begin{array}{l}{\min}_{\mathbf{A},\mathbf{B}}={\left\Vert \mathbf{Y}-{\mathbf{A}\mathbf{B}}^{\mathrm{T}}\right\Vert}_F^2+{\lambda}_l\left({\left\Vert \mathbf{A}\right\Vert}_F^2+{\left\Vert \mathbf{B}\right\Vert}_F^2\right)\\ {}+{\lambda}_d{\left\Vert {\mathbf{K}}_{\mathrm{m}}-{\mathbf{A}\mathbf{A}}^{\mathrm{T}}\right\Vert}_F^2+{\lambda}_t{\left\Vert {\mathbf{K}}_{\mathrm{d}}-{\mathbf{BB}}^{\mathrm{T}}\right\Vert}_F^2,\end{array}} $$

Then, the objective function is further written as

13 $$ {\displaystyle \begin{array}{l}{\min}_{\mathbf{A},\mathbf{B}}={\left\Vert \mathbf{Y}-{\mathbf{A}\mathbf{B}}^{\mathrm{T}}\right\Vert}_F^2+{\lambda}_l\left({\left\Vert \mathbf{A}\right\Vert}_F^2+{\left\Vert \mathbf{B}\right\Vert}_F^2\right)+{\lambda}_d{\left\Vert \alpha {\mathbf{S}}_{\mathrm{m}}+\left(1-\alpha \right){\mathbf{GIP}}_{\mathrm{m}}-{\mathbf{A}\mathbf{A}}^{\mathrm{T}}\right\Vert}_F^2\\ {}+{\lambda}_t{\left\Vert \alpha {\mathbf{S}}_{\mathrm{d}}+\left(1-\alpha \right){\mathbf{GIP}}_{\mathrm{d}}-{\mathbf{BB}}^{\mathrm{T}}\right\Vert}_F^2.\end{array}} $$

More importantly, when predicting novel MDAs, the nearest neighbour information will affect the final results. Therefore, the nearest profile (NP) is introduced to the CMF. For example, the NP for a new miRNA *M*(*i*) is computed as

14 $$ {\mathbf{Y}}_{\mathrm{NP}}\left({M}_i\right)={\mathbf{K}}_{\mathrm{m}}\left({M}_i,{M}_{nearest}\right)\times \mathbf{Y}\left({M}_{nearest}\right), $$

where *M*_*nearest*_ is the miRNA most similar to *M*_*i*_, and **Y**_NP_(*M*_*i*_) is the association profile of miRNA *M*_*i*_. The NP for a new disease *d*_*i*_ is computed as

15 $$ {\mathbf{Y}}_{\mathrm{NP}}\left({d}_i\right)={\mathbf{K}}_{\mathrm{d}}\left({d}_i,{d}_{nearest}\right)\times \mathbf{Y}\left({d}_{nearest}\right), $$

where *d*_*nearest*_ is the disease most similar to *d*_*i*_, and **Y**_NP_(*d*_*i*_) is the association profile of disease *d*_*i*_.

The NP process can be performed in four steps. First, the self-similarity of the matrices **K**_m_ and **K**_d_ is removed. Next, the nearest neighbour of each miRNA and disease is obtained. Then, all miRNA similarities and disease similarities are reset to 0. Finally, the nearest neighbour matrix **N**_m_ of the **K**_m_-based miRNA is obtained. In the previous study [49], the definition of the nearest neighbour matrix is given. According to Eq. (14), we can obtain **N**_m_ = arg max **K**_m_(*M*_*i*_). Simultaneously, the nearest neighbour matrix **N**_d_ of the **K**_d_-based disease is also obtained. According to Eq. (15), we can obtain **N**_d_ = arg max **K**_d_(*d*_*i*_). Based on objective function (11), the objective function of NPCMF can be written as follows:

16 $$ {\min}_{\mathbf{A},\mathbf{B}}={\left\Vert \mathbf{Y}-{\mathbf{A}\mathbf{B}}^{\mathrm{T}}\right\Vert}_F^2+{\lambda}_l\left({\left\Vert \mathbf{A}\right\Vert}_F^2+{\left\Vert \mathbf{B}\right\Vert}_F^2\right)+{\lambda}_d{\left\Vert {\mathbf{N}}_{\mathrm{m}}-{\mathbf{A}\mathbf{A}}^{\mathrm{T}}\right\Vert}_F^2+{\lambda}_t{\left\Vert {\mathbf{N}}_{\mathrm{d}}-{\mathbf{BB}}^{\mathrm{T}}\right\Vert}_F^2, $$

where ‖⋅‖_*F*_ is the Frobenius norm, and *λ*_*l*_, *λ*_*d*_ and *λ*_*t*_ are non-negative parameters. The first term is an approximate model of the matrix **Y**. In the second term, the Tikhonov regularization is used to minimize the norms of **A**, **B**. The last two regularization terms minimize the squared error between **N**_m_ (**N**_d_) and **AA**^T^ (**BB**^T^).

### Initialization of **A** and **B**

For the input MDAs matrix, **A** and **B** are initialized by the singular value decomposition (SVD) method. The initialization formula can be written as follows:

17 $$ \left[\mathbf{U},\mathbf{S},\mathbf{V}\right]=\mathrm{SVD}\left(\mathbf{Y},k\right),\mathbf{A}={\mathbf{US}}_{\mathrm{k}}^{1/2},\mathbf{B}={\mathbf{VS}}_{\mathrm{k}}^{1/2}, $$

where **S**_k_ is a diagonal matrix, which contains the *k* largest singular values.

### Optimization

Considering that the least squares method is an effective way to update **A** and **B**, in this paper, the least squares method is used to update **A** and **B**. **A** and **B** are updated until convergence. *L* is represented as the objection function of the NPCMF method. Then, **A** and **B** are respectively subjected to partial derivatives. *∂L*/*∂***A** and *∂L*/*∂***B** are both set to 0. In addition, *λ*_*l*_, *λ*_*d*_ and *λ*_*t*_ are automatically determined optimal parameter values by the five-fold cross-validation. The update rules are as follows:

18 $$ \mathbf{A}=\left(\mathbf{YB}+{\lambda}_d{\mathbf{N}}_{\mathrm{m}}\mathbf{A}\right){\left({\mathbf{B}}^{\mathrm{T}}\mathbf{B}+{\lambda}_l{\mathbf{I}}_{\mathrm{k}}+{\lambda}_d{\mathbf{AA}}^{\mathrm{T}}\right)}^{-1}, $$

19 $$ \mathbf{B}=\left({\mathbf{Y}}^{\mathrm{T}}\mathbf{A}+{\lambda}_t{\mathbf{N}}_{\mathrm{d}}\mathbf{B}\right){\left({\mathbf{A}}^{\mathrm{T}}\mathbf{A}+{\lambda}_l{\mathbf{I}}_{\mathrm{k}}+{\lambda}_t{\mathbf{B}}^{\mathrm{T}}\mathbf{B}\right)}^{-1}. $$

Therefore, the specific algorithm of NPCMF is as follows:

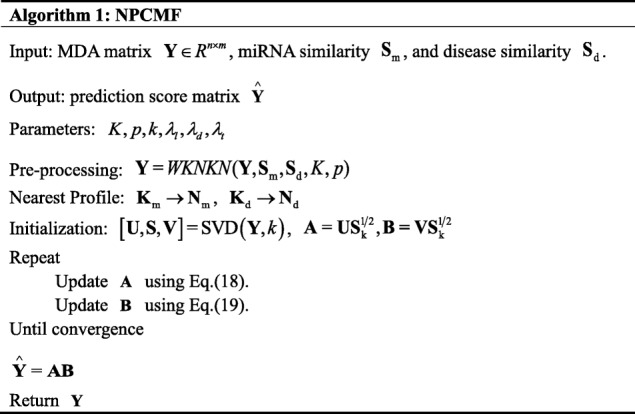



## Data Availability

The datasets that support the findings of this study are available in https://github.com/cuizhensdws/npcmf.

## Abbreviations

CMF: Collaborative matrix factorization method
CV: Cross-validation
NPCMF: Nearest Profile-based Collaborative Matrix Factorization
SVD: Singular value decomposition
WKNKN: Weighted K Nearest Known Neighbours
